# The Role of DCT in HPV16 Infection of HaCaTs

**DOI:** 10.1371/journal.pone.0170158

**Published:** 2017-01-17

**Authors:** Pınar Aksoy, Patricio I. Meneses

**Affiliations:** Department of Biological Sciences, Fordham University, Bronx, New York, United States of America; International Centre for Genetic Engineering and Biotechnology, ITALY

## Abstract

Persistent infection with high-risk human papillomavirus (HPV) genotype is a major factor leading to many human cancers. Mechanisms of HPV entry into host cells and genome trafficking towards the nucleus are incompletely understood. Dopachrome tautomerase (DCT) was identified as a cellular gene required for HPV infection in HeLa cells on a siRNA screen study. Here, we confirm that DCT knockdown significantly decreases HPV infection in the human keratinocyte HaCaT cells as was observed in HeLas. We investigated the effects of DCT knockdown and found that DCT depletion caused increased reactive oxygen species (ROS) levels, DNA damage and altered cell cycle in HaCaT cells. We observed increased viral DNA localization at the endoplasmic reticulum but an overall decrease in infection in DCT knockdown cells. This observation suggests that viral DNA might be retained in the ER due to altered cell cycle, and viral particles are incapable of further movement towards the nucleus in DCT knockdown cells.

## Introduction

Human papillomavirus (HPV) is a non-enveloped small DNA virus. The capsid consists of two virally encoded proteins, L1 and L2 [1, 2]. The L1 protein has been shown to mediate the initial host cell binding at the extracellular matrix or at the plasma membrane [3–5] via the capsid’s interaction with heparan sulfate proteoglycans (HSPGs) [6–8]. After the initial binding event, several conformational changes of the capsid by cellular proteases allow for viral internalization [9–14]. After the virus is internalized into the host cells, the L2 protein, and perhaps L1, accompanies the viral DNA through its journey to the nucleus [15–18]. The viral genome traffics through the endolysosomal sytem, Golgi complex, and the ER before localizing into nucleus during mitosis for viral DNA replication [19–25]. Although we have identified some of the key players in HPV infection, we still lack a complete understanding of this process. Recent genome-wide screening studies provided us with invaluable insights that can help us reveal new players in HPV biology [24, 25].

Dopachrome tautomerase (DCT), also known as tyrosinase-related protein 2, together with tyrosinase (TYR) and tyrosinase-related protein 1 (TRP1) are involved in pigment biosynthesis in mammalian melanocytes [26]. During melanin synthesis, DCT converts L-DOPAchrome to 5,6-dihydroxyindole-5-carboxylic acid (DHICA) [27, 28]. DCT matures in the ER in the presence of calnexin, until it reaches a dithiothreitol-resistant conformation that enables the protein to leave the ER and localize to Golgi. Inhibition of calnexin association of DCT leads to proteasomal degradation of the protein, which implies that misfolded protein is able to exit the ER, localize to the cytosol and be degraded by the proteasome [29]. Immunofluorescence experiments in mouse melanoma cells have showed strong colocalization of DCT and trans-golgi network (TGN) and limited colocalization of DCT with plasma membrane. Because DCT lacks the di-leucine motif that TYR and TRP1 have at their C-terminus, its localization and trafficking differ: it is not solely confined to melanosomes, it can localize to Golgi or plasma membrane, thus suggesting a function for the protein unrelated to melanin biosynthesis [29, 30]. DCT, TYR and TRP1 are all type-I transmembrane proteins that are cleaved by γ-secretase complex [31]. The cleavage has been shown to impact intracellular localization of these proteins, but it is not known whether γ-secretase cleavage of these proteins affects their functionality [32].

DCT was identified in Lipovsky and colleague’s siRNA screen as one of the crucial genes for HPV infection in HeLa cells [25]. In the same screening study, subunits of the γ-secretase complex were also strong hits, which supports earlier understanding of γ-secretase’s importance in HPV infection [22, 33, 34]. The study also recovered other known substrates of γ-secretase complex (such as EPHA4, DSG2, JAG2, BTC, LRP2, PTPRK and ROBO1) and DCT was the only substrate that ranked in the first percentile [25].

Here, we investigated the effects of DCT knockdown in HPV16 pseudovirus (PsV) infection of basal keratinocytes (HaCaT cells). Because the importance of γ-secretase function in HPV infection is well-known and there is evidence that DCT is a substrate of γ-secretase, we also compared the effect of DCT depletion to γ-secretase inhibition on HPV infection. Under DCT depletion, we observed decreased HPV16 pseudovirus (PsV) infection and increased levels of PsVs in the ER. Furthermore, our results suggested that there was altered cell cycle progression in DCT knockdown cells, possibly contributing to the decrease in HPV16 PsV infection. Finally, DCT depleted and γ-secretase inhibited cells showed similar yet not completely identical characteristics during HPV infection.

## Materials and Methods

### Cell culture and HPV16 PsV production

HaCaT cells (in vitro spontaneously transformed keratinocytes from histologically normal skin) were purchased from AddexBio (San Diego, CA). Cells were cultured in Dulbecco's Modified Eagle's media (DMEM, Thermo Scientific, Waltham, MA)) supplemented with 10% Fetal Bovine Serum, DMEM-10 (FBS, Gemini Bio-products, West Sacramento, CA). HPV16 pseudovirion (PsV) production and purification were performed as previously described [19, 35]. Briefly, 8fwb (GFP expressing plasmid) and p16sheLL (HPV16 L1 and L2 plasmid) were transfected into 293TT cells. Cells were harvested and after high salt extraction, PsV were purified on an optiprep gradient (27–39%). Alternatively, 6 hours after DNA transfection of 293TT cells, growth medium was supplemented with 10μM 5-Ethynyl-2´-deoxyuridine (EdU; C10340, Life Technologies, Norwalk, CT) to generate EdU-labeled pseudogenome harboring HPV16 PsV. In our experiments, infection is detected by the expression of GFP using flow cytometer. (BD Accuri C6 flow cytometer, Franklin Lakes, NJ). The PsV preparation we used had a titer of 10^7^ (10,000 transducing units per microliter). We infected the cells to achieve 15% GFP expression. Based on our q-PCR (Applied Biosystems, Life Technologies) this roughly corresponds to 600 plasmid carrying particles per cell (or viral genome equivalent). 293TT cells were a generous gift from Dr. Ozbun (The University of New Mexico School of Medicine, NM).

### siRNA-mediated knockdown of DCT and infection

DCT knockdown was performed as previously described [36]. To target human DCT gene, we used siRNA sequences based on Michard et. al.’s study: AACCAGUGAUUCGGCAGAACA (sense), UGUUCUGCC GAAUCACUGGUU (anti sense) from Sigma. Cells were plated on 12-well plates at 50% confluency. The next day siRNA transfection was performed at 20nM in Lipofectamine RNAiMAX transfection reagent (Life Technologies) and Opti-MEM reduced serum media (Life Technologies). Non-targeting, control siRNA was purchased from IDT DNA technologies CGUUAAUCGCGUAUAAUACGCGUAT (sense), AUACGCGUAUUAUACGCGAUUAACGC (anti sense) (Coralville, IA). The following day, a second siRNA transfection was performed on the cells under same conditions. 72 hours after first transfection, cells were counted and replated at 50% confluence and infected with PsV on the following day as described above. After infection, the media was aspirated to remove any unbound virus, and cells were washed three times with PBS. After 48 hours at 37°C, cells were harvested by trypsinization and infection levels were detected with flow cytometer as described above. On the day of infection, cell lysates were also harvested in lysis buffer to check the siRNA efficiency (Nonidet P-40, United States Biological, Salem, MA, and 50X protease inhibitor cocktail, Promega, Madison, WI). Retinal pigment epithelium (RPE) cell lysate was a kind gift of Dr. Finnemann (Fordham University, Bronx, NY). Cell lysates were run on a 10% SDS-PAGE and proteins were transferred onto nitrocellulose membrane (Life Technologies). Membranes were blocked with 5% non-fat dry milk for 1 hour and then incubated with anti-DCT mouse antibody (sc-74439, 1:500 dilution) (Santa Cruz Biotechnology, Dallas, TX) or anti-actin mouse antibody (Sigma A4700, 1:1000 dilution) (Sigma-Aldrich, St. Louis, MO) overnight. After primary antibody incubation, LI-COR secondary antibodies were used at 1:30,000 dilution for 30 minutes (LI-COR, Lincoln, NE). Odyssey Imaging System (LI-COR) was used to scan the membrane and band intensities were analyzed using the built-in software.

### Titration of the γ-secretase inhibitor XXI

γ-secretase inhibitor (Compound E, referred to as XXI in this manuscript) was purchased from EMD Millipore (Billerica, MA) and dissolved in DMSO (Sigma-Aldrich, St. Louis, MO). We started testing different concentrations of XXI, 200 nM-3.2 μM (dilution factor 1:2), and 1 nM-250 nM (dilution factor 1:3), and 1 pM-900 pM (dilution factor 1:3). To test the effect of different concentrations of XXI on HPV16 PsV infection, we incubated cells on ice for 1 hour to slow down endocytosis, and prepared the DMEM-10 containing either XXI in DMSO or DMSO as control. After 1 hour of ice incubation, the media was washed off and DMEM-10 containing either XXI or DMSO was added to the wells. Following this, the cells were infected with HPV16 PsV and kept on ice for another 2 hours. After the ice incubation, the plates were directly put in the 37°C incubator for 48 hours. The cells were then collected via trypsinization (Corning, Manassas, VA) and washed three times with PBS (MP biomedicals, Solon, OH) by spinning at 3000 x g for 5 min. Percentage of infected HaCaT cells was calculated based on the number of GFP positive cells as measured by flow cytometer.

### Antibodies for cellular markers

After the confirmation of DCT knockdown, control siRNA and DCT siRNA transfected cell lysates were subjected to western blotting with the following primary antibodies from Cell Signaling Technology, Beverly, MA: anti-pRb Ser807/ 811 rabbit antibody (8516, 1:1000 dilution), anti-PARP1 rabbit antibody (9542, 1:1000 dilution), anti-Caspase3 rabbit antibody (9662, 1:1000 dilution), anti-cleaved Caspase3 rabbit antibody (9664, 1:1000 dilution), anti-pChk2 Thr68 rabbit antibody (2197, 1:500 dilution), total Chk2 rabbit antibody (2662, 1:1000 dilution) and anti-pH2A.X Ser139 rabbit antibody (2577, 1:1000 dilution).

### Binding assay

DCT or control siRNA treated cells were replated on a 6-well plate at 50% confluence 72 hours after first siRNA transfection. On the day of infection, DCT knockdown and control cells were counted and infected. The cells were kept on ice for 1 hour to slow down endocytosis. Following this, the cells were infected with HPV16 PsVs and kept on ice for 1 hour to facilitate viral binding. For DMSO or XXI treatment, 300,000 HaCaT cells were plated on a 6-well-plate the day before infection. The next day, the cells were kept on ice for 1 hour and after the ice incubation they were treated with DMSO or XXI at 300pM. Following this, the cells were infected with HPV16 PsVs. Infected cells were placed on ice for 1-hour incubation to facilitate viral binding. Excess virus was washed off and the cell lysates were harvested in lysis buffer as described above. The cell lysates were run on an 8% SDS-PAGE. After the western blotting and blocking, the nitrocellulose membranes were incubated with anti-HPV antibody (BPV-1/1H8 + CAMVIR, ab2417; Abcam, Cambridge, United Kingdom) and actin antibody at 1:1000 dilution.

### Immunofluorescence experiments

#### Early endosome localization

DCT or control siRNA treated cells were replated on two 12-well plates, containing glass coverslips, at 50% confluence 72 hours after first transfection. For DMSO or XXI treatment, 100,000 HaCaT cells were plated on two 12-well-plates so that they were at 50% confluence on the day of infection. Viral binding was performed as described above. Excess virus was washed off for control or DCT siRNA treated cells and the plates were incubated in 37°C incubator for 4 hours. For DMSO or XXI treated cells, the plates were directly placed in 37°C incubator for 4 hours without washing off the DMSO or XXI treatment and the viral particles. After incubation time, the cells were washed three times with PBS and fixed with 4% paraformaldehyde (PFA) for 10 minutes on ice and washed three times with PBS. Cells were permeabilized using blocking buffer (0.2% fish skin gelatin Sigma, 0.2% Triton X- 100 Sigma in PBS) for 1 hour at room temperature. The cells were then incubated with corresponding primary antibodies for 1 hour: mouse antibody H16.V5-against HPV16 PsV, 1:100 dilution (kind gift from Dr. Christensen, Penn State Hershey Medical Center, Hershey, PA) and goat antibody EEA1-early endosome marker, 1:100 dilution, (sc-6414, Santa Cruz Biotechnology). The coverslips were washed three times with PBS and incubated with secondary antibodies (Alexa-Fluor donkey anti-mouse 647, and anti-goat 568) at 1:2,000 dilutions. Antibody dilutions were prepared in blocking buffer. After secondary antibody incubation, the coverslips were washed three times with PBS and mounted on slides using ProLong Gold Antifade Reagent with DAPI. Alexa antibodies and prolong with DAPI were purchased from Life Technologies. Images were acquired on a Leica TSP5 (Leica, Mannheim, Germany) laser-scanning confocal microcopy system at Fordham University (Bronx, NY).

#### Trans-Golgi network (TGN) localization

After 16 hours at 37°C incubator, the cells were fixed with 4% PFA. For the samples that were infected with non-EdU pseudovirus, coverslips were permeabilized using the blocking buffer for 1 hour. The coverslips were then incubated with corresponding primary antibodies for 1 hour: H16.V5 (1:100 dilution) and TGN-46 (sheep, 1:100, AbD Serotec, Raleigh, NC). Alexa-Fluor donkey anti-mouse 647 and anti-sheep 568 were used as secondary antibodies. For pseudovirus carrying EdU-labeled pseudogenome, the coverslips were first treated with Click-iT reaction cocktail at 647nm (Life Technologies) for 30 minutes to visualize the EdU-labeled pseudogenome, and washed once with PBS. The cells were then blocked with the blocking buffer for 1 hour and TGN staining was performed as described above.

#### Endoplasmic reticulum (ER) localization

The cells were incubated for 12, 16, or 20 hours at 37°C incubator. To visualize the ER, anti-GRP78 BiP antibody was used at 1:100 dilution (ab21685, Abcam). Alexa-Fluor donkey anti-rabbit 568 was used at 1:2,000 dilution.

#### Reactive oxygen species (ROS) detection

DCT or control siRNA treated cells were replated on a 12-well plate, containing glass coverslips, at 50% confluence 72 hours after first transfection. The next day, 5μM CellROX deep red reagent (Life Technologies) was added to the wells. Cells were incubated with the reagent for 30 minutes at 37°C. The cells were washed three times with PBS and fixed with 4% PFA and washed three times with PBS. The coverslips were mounted on microscope slides with ProLong Gold Antifade Reagent with DAPI and immediately scanned by the confocal microscope. For the detection of ROS under DMSO or XXI treatment, 150,000 HaCaTs were plated on a 12-well plate with coverslips. The cells were treated with DMSO and 300pM XXI, and they were incubated for 4 hours or 24 hours at 37°C together with the treatments. After that, 5μM CellROX deep red reagent was directly added to the wells and the cells were fixed and scanned as described above.

### Colocalization analysis

The confocal images were processed by ImageJ and quantitatively analyzed by JaCoP plug in to measure M1 (Manders) coefficient, i.e., percentage of virus (red) signal overlapping with organelle (green) signal. Analysis was performed on the center slice of at least three separate Z-stacks scanned from each coverslip. Representative data are shown from three independent experiments (a total of nine scans).

### Cell cycle analysis by flow cytometry

DCT and control siRNA transfected cells were harvested by trypsinization five days after the first siRNA transfection. To achieve equal amounts of propidium iodide (PI) staining, the amount of PI added to each sample was adjusted according to the number of cells in a sample (1 μl PI solution per 1,000 cells). DMSO and XXI treated cells were harvested by trypsinization one or two days after treatment. For PI staining, the cells were centrifuged and the cell pellet was washed three times with PBS. Then, the cells were fixed with ethanol and kept at -20°C overnight. The next day fixed cells were centrifuged and washed twice with PBS. Then, the cells were stained with PI staining solution for 20 minutes at 37°C. PI staining solution: 1% (v/v) Triton X-100, 400μl of 0.5mg/ml PI Roche (Indianapolis, IN), 84μl of 24mg/ml RNase A (Roche) in 10ml PBS. Cell cycle profiles were detected by flow cytometer and the data were analyzed by FlowJo version 10, cell cycle model: Watson (pragmatic).

## Results

### DCT siRNA-mediated knockdown results in decreased HPV16 PsV infection in HaCaTs

DCT is a γ-secretase substrate shown to be one of the top ten hits in a genome-wide siRNA screen that identified cellular genes needed for HPV infection in HeLa cells [32, 37]. Inhibition of γ-secretase by XXI has been shown to reduce HPV16 PsV infection in HeLa and HaCaT cells [22, 34]. We wanted to explore the roles of DCT in HPV16 infection and compare the findings to the requirement of γ-secretase action during HPV16 infection. We began by confirming that γ-secretase inhibition with the inhibitor XXI reduces HPV16 PsV infection in HaCaT cells. We determined that treatment with 300pM XXI led to a complete loss of HPV16 PsV infection as measured by flow cytometry analysis of GFP expressing cells (S1 Fig). Lower concentrations of XXI, at 100pM, 30pM, 10pM decreased infection by 71%, 21%, and had no effect on infection levels respectively (S1 Fig).

**Fig 1:**
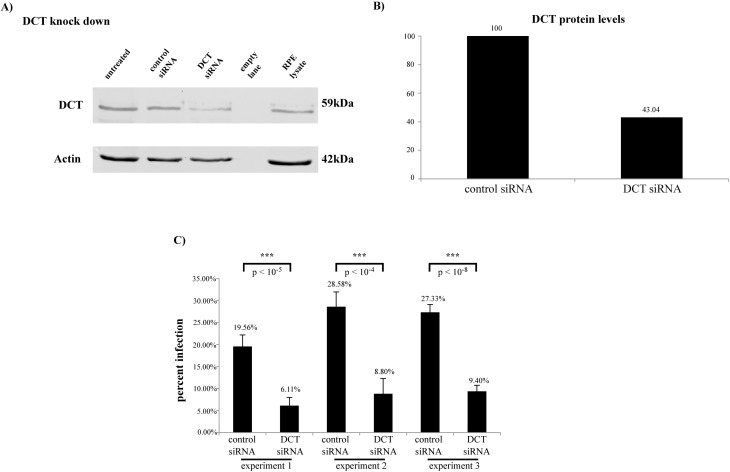
DCT depletion leads to a significant decrease in HPV infection in HaCaTs. (A) DCT antibody incubation shows the partial DCT knockdown in DCT siRNA treated cells. Actin was used as the loading control. (B) DCT band intensity for control and DCT siRNA treated cells was normalized to corresponding actin measurements. DCT protein levels decreased by 57% in DCT siRNA treated cells. (C) HPV16 PsV infection significantly decreased in DCT knockdown HaCaTs in three independent experiments compared to control siRNA treated cells (two-tailed t-test, p-values: 0.00000135, 0.000017 and 0.0000000022). Infection was quantified by flow cytometry. Error bars show the standard deviation of six experimental replicates in which 10,000 cells were analyzed for GFP expression to obtain the percent of infected cells.

We addressed DCT’s role in HPV16 PsV infection in HaCaTs. DCT siRNA transfection of HaCaTs resulted in a 57% decrease in DCT protein level (Fig 1A and 1B) and reduced HPV16 PsV infection by 68% (Fig 1C; three independent experiments shown). Retinal pigment epithelium (RPE) lysate was included as a positive control for DCT expression and detection. These experiments showed that DCT knockdown reduced HPV16 PsV infection in HaCaTs, and that expression level of DCT in HaCaTs was comparable to its expression level in RPE (DCT protein level normalized to actin: 0.42 in HaCaTs and 0.23 in RPE, Fig 1A).

### DCT depletion does not interfere with viral binding and initial endocytosis of the virus

**Fig 2:**
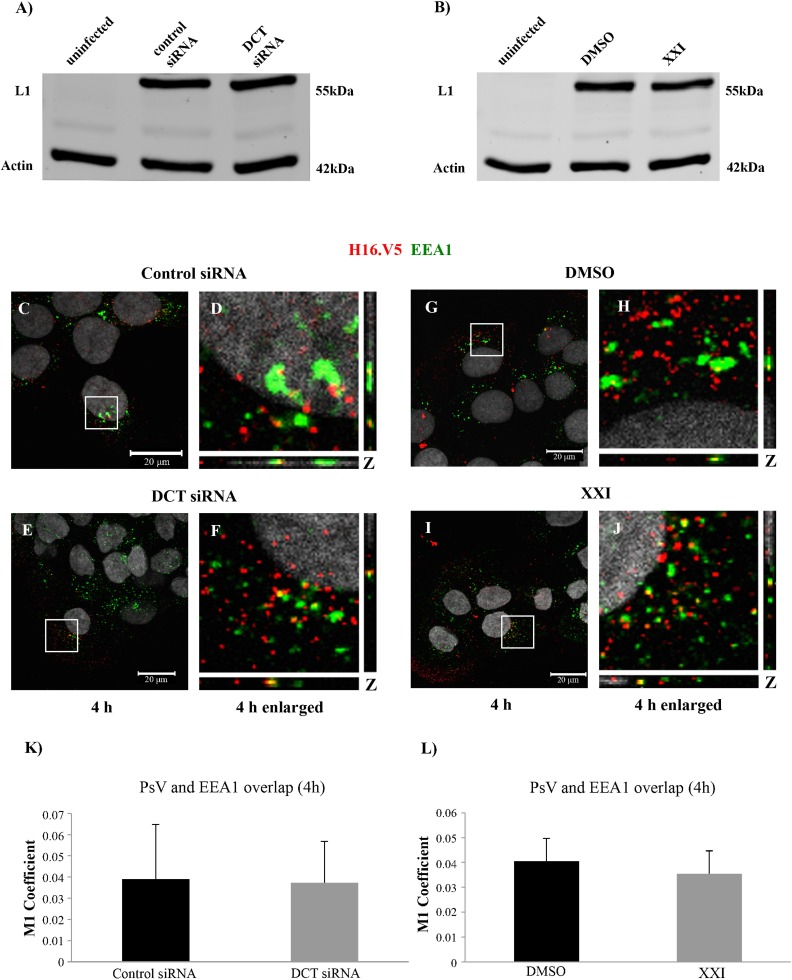
DCT siRNA or XXI treatment does not interfere with binding and early endosome localization of HPV16 PsV in HaCaTs. Western blot analysis of L1 and actin levels in (A) uninfected, control and DCT siRNA transfected HaCaTs, (B) uninfected, DMSO and 300pM XXI treated HaCaTs. (C-J) Immunofluorescence analyses of EEA1 (green), L1 (red) in control and DCT siRNA transfected and DMSO and XXI treated cells. Nuclei are stained with DAPI (grey). Colocalization of L1 and EEA1 appears yellow. (K and L) The JACoP plugin for ImageJ was used to measure the M1 coefficient (fraction of red signal overlapping with green signal) with three confocal scans for each condition.

To determine where DCT knockdown interferes in the infectious process, we first examined binding and internalization of PsVs. We infected control or DCT siRNA transfected cells, and kept the cells on ice to facilitate viral binding. We washed off unbound viral particles and harvested the cell lysates. Based on our binding assay results by western blot, we observed comparable levels of cell-bound L1 protein in control and DCT siRNA transfected cells (Fig 2A). Cell-bound L1 protein levels were also similar in DMSO and XXI treated cells (Fig 2B). Our results suggested that viral binding was not affected by DCT knockdown or XXI treatment.

After binding and endocytosis, the viral particles have been shown to localize to early endosomes [19]. To follow viral trafficking under different treatments, we used confocal microscopy and antibodies against the viral capsid (H16.V5) and early endosome marker (EEA1). HPV16 PsVs were found to localize to early endosomes at 4 hours post infection (hpi) at comparable levels in DCT knockdown and control cells as detected by the overlap of the green (EEA1) and red (H16.V5) signal (Fig 2C–2F and 2K). As a comparison, we performed the same experiment in XXI and DMSO treated cells. We detected comparable levels of endosome localization under both treatments via confocal microscopy (Fig 2G–2J and 2L). These results suggested that the decrease in HPV16 PsV infection was not a result of deficiency in cell binding or early endosomal internalization of the viral particles in DCT siRNA treated cells.

### HPV16 localizes to the Golgi complex in DCT depleted cells

**Fig 3:**
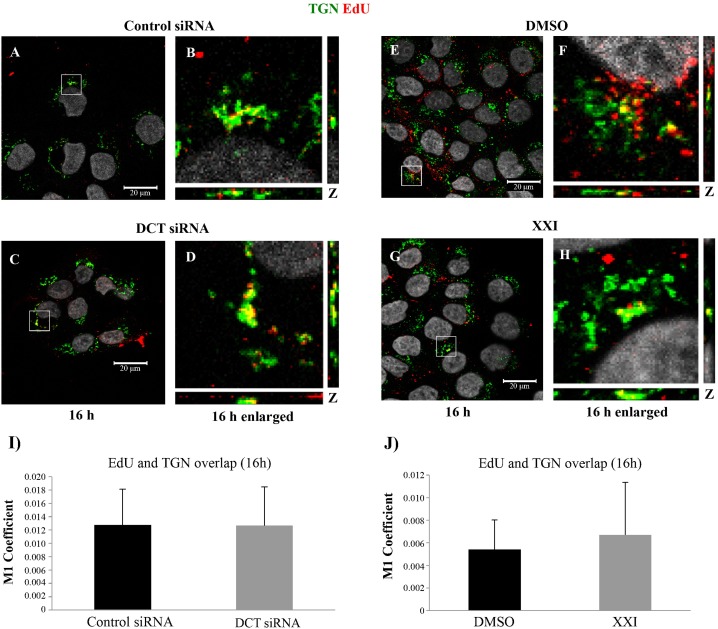
Colocalization of TGN with EdU-labeled pseudogenome is not affected by DCT siRNA or XXI treatment of HaCaTs at 16hpi. (A-H) control siRNA, DCT siRNA, DMSO or XXI, treated HaCaTs were infected with HPV16 PsV harboring EdU-labeled pseudogenome. TGN was stained with TGN-46 (green), EdU-labeled pseudogenome was visualized by Click-iT reaction (red) and the nuclei were stained with DAPI (grey). Colocalization of EdU and TGN appears yellow (I and J) The JACoP plugin for ImageJ was used to measure the M1 coefficient (fraction of red signal overlapping with green signal) with three confocal scans for each condition.

HPV16 PsVs have been shown to traffic from the endosomal system to the trans-Golgi network (TGN) during infection [21, 37, 38]. Several studies suggest that during endosomal trafficking, as the pH decreases in late endosomes/lysosomes, the viral capsid disassembles and majority of the L1 proteins are degraded, whereas L2/viral DNA (vDNA) traffics towards the TGN. Based on these, we wanted to investigate whether viral particles localized to Golgi complex in DCT depleted cells. For this, we detected the viral pseudogenome via EdU-labeling and Click-iT reaction and used TGN46 antibody to visualize the TGN. Immunofluorescence assays at 16hpi showed similar levels of colocalization of the pseudogenome and TGN46 in control versus DCT siRNA transfected HaCaTs (Fig 3A–3D and 3I). Similarly, we observed comparable levels of TGN localization in XXI and DMSO treated cells (Fig 3E–3H and 3J).

We also checked viral localization to Golgi complex with H16.V5 (against major capsid protein, L1) and TGN46 antibodies at 16hpi. We observed similar levels of colocalization of L1 and TGN46 in control versus DCT siRNA transfected HaCaTs, or DMSO versus XXI treated (S2A–S2J Fig). These results suggested that some L1 particles were able to localize to the TGN, probably together with L2/vDNA complex, and viral trafficking to the TGN was not affected in DCT knockdown or XXI treated cells.

### Endoplasmic reticulum localization of the viral pseudogenome is increased in DCT knockdown HaCaTs

It has been shown that after the Golgi complex, the viral particles localize to the ER. In our previous studies, we reported PsV trafficking to the ER using the H16.V5 antibody and two ER markers (ERp29 and calnexin), and Zheng and colleagues showed HPV16 PsV in the ER during trafficking, using a FLAG-tagged L2 and two ER markers (BiP and PDI) [22, 23]. Because the viral particles were able to localize to the TGN under DCT siRNA transfection, we wondered if they would localize to the ER.

**Fig 4:**
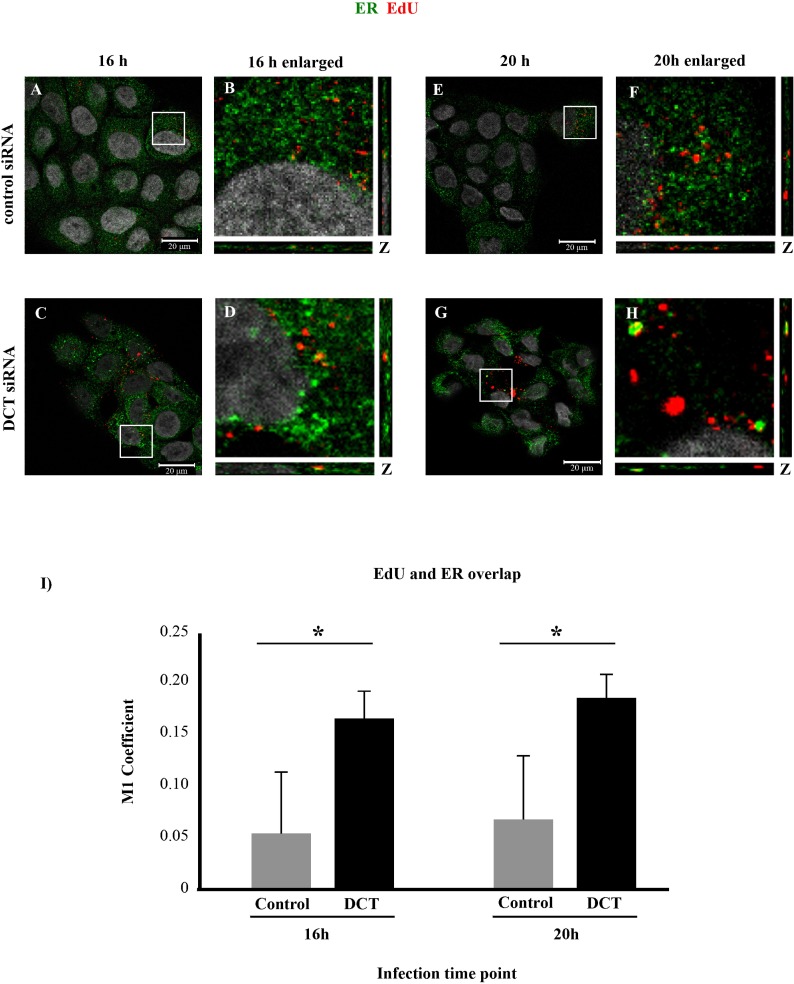
DCT knockdown causes increased ER trafficking of EdU-labeled pseudogenome. DCT or control siRNA treated cells were infected with HPV16 PsV harboring EdU-labeled pseudogenome for 16 h (A-D) or 20 h (E-H). ER was stained with BiP (green), EdU-labeled pseudogenome was visualized by Click-it reaction (red) and the nuclei were stained with DAPI (grey). (A-D and I) Control siRNA treated HaCaTs showed almost no colocalization, while significantly higher levels of colocalization was observed in DCT siRNA treated cells at 16hpi (one-tailed t-test p = 0.015). (E-H) At 20hpi, the levels of colocalization were still significantly higher in DCT siRNA treated cells compared to control cells (one-tailed t-test p = 0.018). (I) M1 coefficient, percentage of red signal (EdU-labeled pseudogenome) overlapping with green signal (BiP) was calculated with the help of JaCOP plug in with nine confocal scans for each condition. (A-H). Error bars show the standard deviation in measurements from three independent replicates.

We performed ER immunofluorescence experiment in DCT knockdown and control cells at 16 hpi and observed significantly higher levels of colocalization of the ER marker and EdU-labeled pseudogenome in DCT knockdown cells compared to the control cells (Fig 4A–4D and 4I, one-tailed t-test p = 0.015). This result may suggest that the kinetics of infection under DCT depletion is different than control cells, so the virus may traffic to the ER earlier in DCT depleted cells. To test this possibility, we repeated the same immunofluorescence analysis at a later time point, 20hpi. Colocalization of EdU-labeled pseudogenome and BiP was again significantly higher in DCT knockdown cells compared to control cells (Fig 4E–4H and 4I, one-tailed t-test p = 0.018).

**Fig 5:**
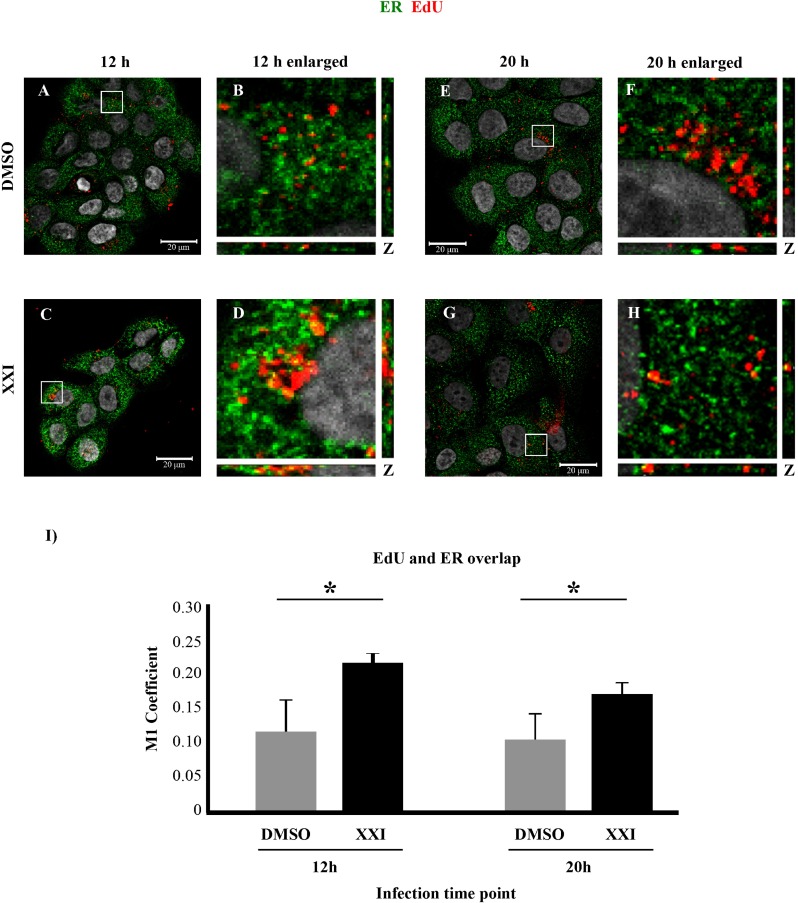
γ-secretase inhibition causes increased ER trafficking of EdU-labeled pseudogenome. DMSO or XXI treated cells were infected with HPV16 PsV harboring EdU-labeled pseudogenome for 12 h (A-D) or 20 h (E-H). ER was stained with BiP (green), EdU-labeled pseudogenome was visualized by Click-it reaction (red) and the nuclei were stained with DAPI (grey). (A-D and I) DMSO treated HaCaTs showed significantly less colocalization compared to XXI treated HaCaTs at 12hpi (one-tailed t-test p = 0.008). (E-H and I) At 20hpi, significantly higher levels of colocalization were detected in XXI treated cells compared to DMSO treated cells (one-tailed t-test p = 0.02). (I) M1 coefficient, percentage of red signal (EdU-labeled pseudogenome) overlapping with green signal (BiP) was calculated with the help of JaCOP plug in with nine confocal scans for each condition. (A-H). Error bars show the standard deviation in measurements from three independent replicates.

Furthermore, we found that EdU-labeled pseudogenome colocalized with ER marker BiP in DMSO or XXI treated cells at 12hpi, with more colocalization observed under XXI treatment (Fig 5A–5D and 5I, one-tailed t-test p = 0.008). To investigate the same question about a possible change in kinetics of infection in XXI treated cells, we repeated the immunofluorescence analysis at a later time point, 20 hpi. We observed that colocalization of EdU-labeled pseudogenome and the ER marker was again significantly higher in XXI treated cells (Fig 5E–5H and 5I, one-tailed t-test p = 0.02).

Our results show that viral particles were able to traffic to the ER in DCT knockdown and XXI treated cells. Although ER trafficking was not impaired, infection in in DCT knockdown cells was either significantly decreased compared to the control cells or completely inhibited in the case of XXI treated cells.

### DCT depletion causes increased ROS levels, DNA damage and altered cell cycle in HaCaT cells

DCT overexpression has been shown to reduce cellular sensitivity to oxidative stress and protect the DNA from oxidative stress damage in WM35 melanomas [36]. It is known that oxidative DNA damage can lead to cell cycle arrest; and HPV requires nuclear envelope breakdown during mitosis to be able to gain entry into the nucleus [24, 39, 40]. Along these lines, we consistently observed a decrease in population in the DCT knockdown cells compared to the control siRNA transfected cells (S1 Table). We wanted to determine whether DCT knockdown causes oxidative stress in HaCaT cells, which, in turn, might be leading to increased DNA damage and/or cell cycle arrest.

**Fig 6:**
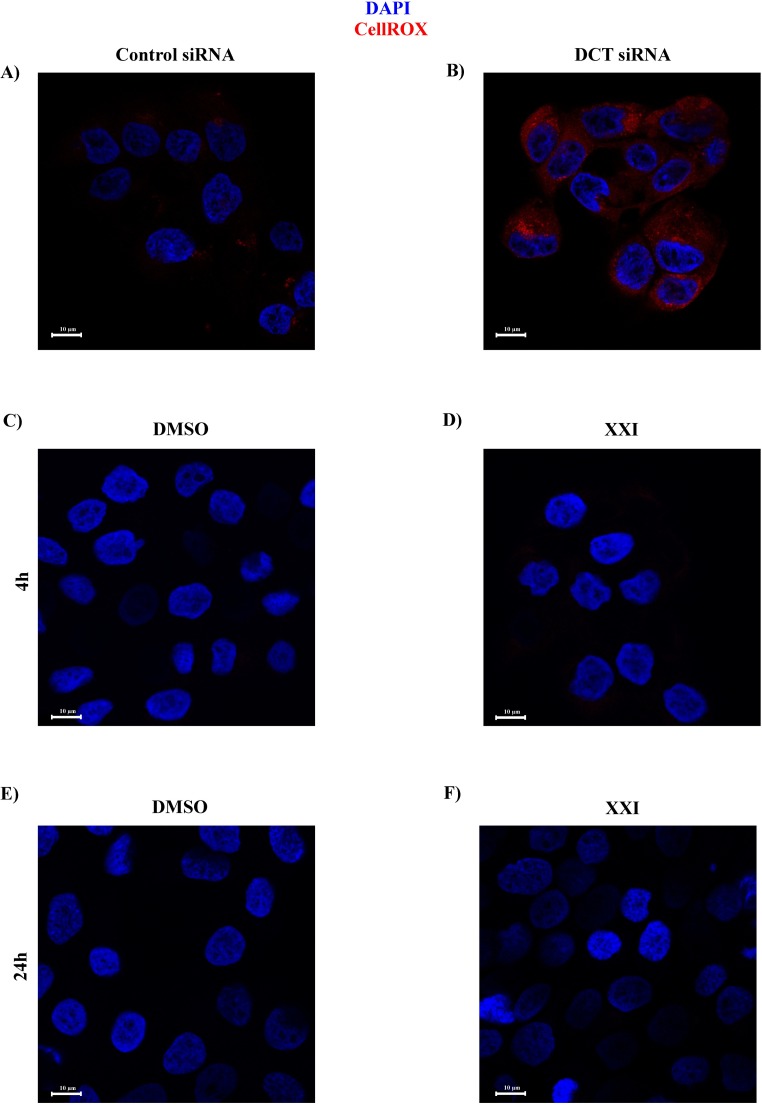
Depletion of DCT causes increased ROS production, whereas XXI treatment does not. On the day of the knockdown, control and DCT knockdown cells were treated with CellROX deep red reagent to detect oxidative stress levels under each treatment. The cells were fixed and visualized by confocal microscopy. Cell nuclei were stained with DAPI (blue). Oxidized CellROX signal was detected in red. (A) Control HaCaTs. (B) DCT knockdown HaCaTs. (C-F) HaCaTs were treated with XXI or DMSO for 4h or 24h and CellROX reagent was added to detect cellular ROS.

To measure the oxidative stress, we treated the DCT knockdown and control cells with CellROX deep red reagent on the day of knockdown (96 hours after first round of siRNA transfection) and investigated the cells under confocal microscopy. The CellROX reagent is non-fluorescent while at a reduced state but becomes fluorescent upon its oxidation by cellular reactive oxygen species (ROS). Unlike control cells, we saw a prominent red signal in DCT knockdown cells using confocal microscopy (Fig 6, compare B to A). This indicated that DCT knockdown cells had higher levels of ROS compared to the control cells.

We also wanted to determine whether XXI treatment caused ROS in HaCaTs, although we never observed low cell counts in XXI treated cells compared to DMSO treated cells. Unlike the DCT knockdown cells, we did not detect ROS in either DMSO or XXI treated cells following 4 and 24 hours of treatment with the corresponding reagent (Fig 6C–6F). These results suggested that oxidative stress was not a contributor to the block in infection in XXI treated cells. For the rest of the study, we investigated the mechanism of decrease in infection in DCT knockdown cells.

**Fig 7:**
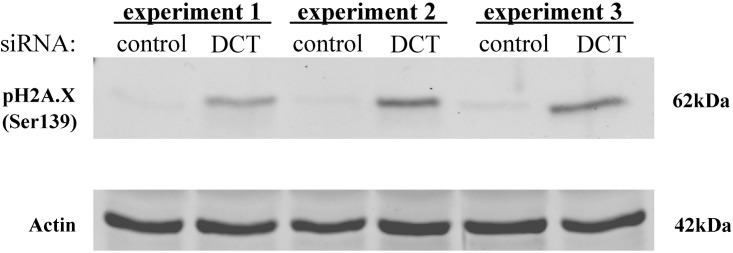
There is increased DNA damage in DCT knockdown cells compared to control cells. Cell lysates from control and DCT knockdown cells were harvested and subjected to western blotting on the day of the knockdown. Western blot analysis of pH2A.X phosphorylated at Ser139 (DNA damage marker) and actin (loading control) are shown for three independent DCT knockdown experiments.

An increase in the cellular ROS level has been shown to cause cellular DNA damage [40]. Because we observed higher levels of ROS in DCT knockdown cells compared to control cells, we wanted to check the degree of DNA damage in these cells. H2A.X is a histone 2A variant and it becomes phosphorylated at Ser139 when there is DNA-damage and this phosphorylated state recruits DNA-damage response proteins to the site of damage [41]. To detect DNA damage in DCT knockdown cells, we checked the cellular levels of this damage marker via pH2A.X (Ser139) antibody. pH2A.X protein was present in DCT knockdown cell lysates, whereas it was barely detectable in control cells (Fig 7). The activation of the DNA damage response pathway arrests the cell cycle to allow time for DNA repair [42]. One of the downstream proteins in the pathway is checkpoint kinase, Chk2. Upon DNA damage; Chk2 is phosphorylated at several serine/threonine residues with Thr68 being the primary site that gets phosphorylated in response to DNA damage [43]. The western blot results with pChk2 were also consistent with our previous findings: there was relatively more pChk2 protein in DCT knockdown cells compared to control cells (S3 Fig).

**Fig 8:**
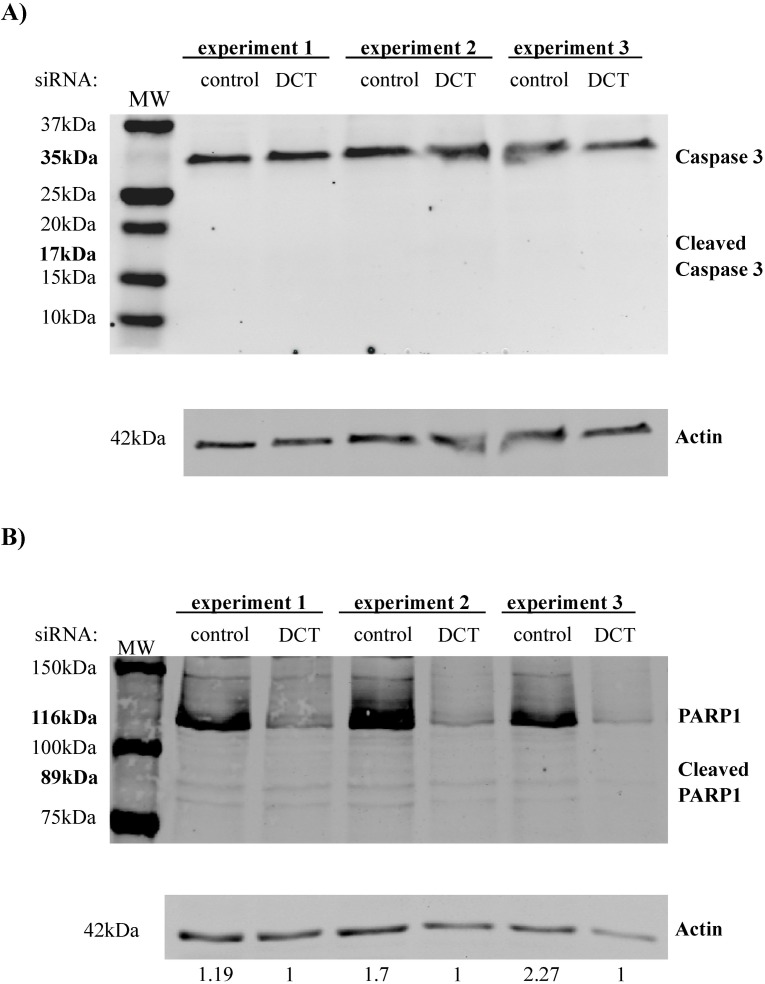
Apoptosis markers are not present in control and DCT knockdown cells. Cell lysates from control and DCT knockdown cells were harvested and subjected to western blotting on the day of the knockdown for three independent DCT knockdown experiments. For none of the samples, cleaved forms of caspase 3 or PARP1 was detectable. (A) Western blot for Caspase 3, cleaved caspase 3 and actin (loading control). (B) Western blot for PARP1, cleaved PARP 1 and actin (loading control).

Our data showed that there was an increase in the ROS levels and DNA damage in DCT knockdown cells, so we next assessed if these changes would cause apoptosis in DCT knockdown cells. For this, we used well-known cellular markers of apoptosis: cleaved caspase 3 and cleaved PARP1. The caspase 3 cleavage product was not observed in any of the treatments (Fig 8A). Total caspase 3 levels were comparable for all of the treatments (Fig 8A). Similarly, the PARP1 cleavage product was not observed in any of the conditions (Fig 8B). These results indicate that DCT depletion did not cause increased apoptotic death of the cells.

**Fig 9:**
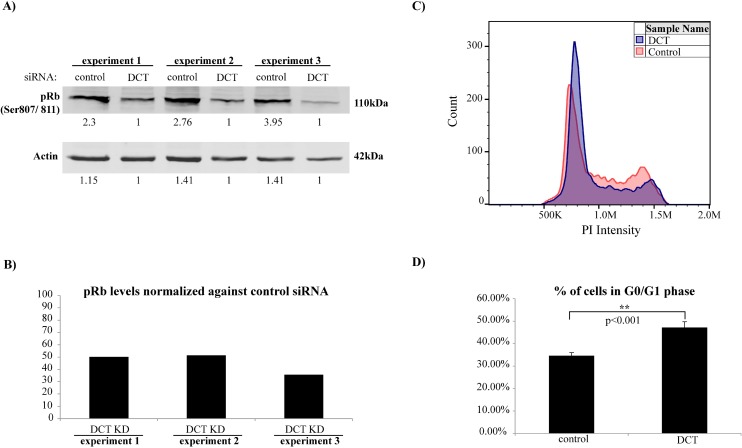
Cell cycle progression is altered in DCT knockdown cells compared to control cells. (A) Cell lysates from control and DCT knockdown cells were harvested and subjected to western blotting on the day of the knockdown. pRb (Ser807/811) and actin levels are shown for three independent DCT knockdown experiments. (B) pRb band intensities of each lane were quantified and normalized first against the corresponding actin measurements and then against the normalized pRb levels in control samples. The bar graph shows the normalized pRb levels of DCT knockdown cells compared to their controls. (C) Representative cell cycle profiles for DCT knockdown and control cells as analyzed and visualized by FlowJo v10. (D) Cell cycle profiles of DCT knockdown and control cells were analyzed by PI staining and flow cytometer. The bar graph shows the mean value of the percentage of cells in G0/G1 phase for three independent experiments with six replicates each (one tailed t-test p = 0.0008).

We next hypothesized that increased levels of ROS and increased DNA damage in DCT knockdown cells would result in cell cycle arrest. To assess the cell cycle progression of DCT knockdown and control cells, cell lysates were subjected to western blotting with pRb (Ser807/811) antibody. Retinoblastoma (Rb) protein is hypophosphorylated at the G0 phase, whereas it becomes phosphorylated during G1 and it remains hyperphosphorylated until the late stages of mitosis. When it is hypophosphorylated, Rb suppresses transcription factors that are needed for the expression of genes that encode products necessary for S phase progression. Once it is hyperphosphorylated, pRb can no longer associate with those transcription factors; therefore, cell cycle progresses into the S phase [44]. Cell lysates from three independent DCT knockdown experiments were subjected to western blotting together with their control cell lysates. The amount of pRb was found to be less in DCT knockdown cells compared to control cells, where DCT knockdown cells had ~55% less pRb protein compared to their controls (Fig 9A and 9B). These data suggested that cell cycle progression might be altered in DCT knockdown cells. Therefore, we wanted to check the cell cycle profile of DCT knockdown and control cells by propidium iodide (PI) staining of DNA and detected the fluorescence signal by flow cytometry. We estimated the fraction of cells that are in the G0/G1 phase by using FlowJo’s cell cycle module (version 10, model:Watson pragmatic). The percentage of cells that were in the G0/G1 phase was significantly higher in DCT knockdown cells compared to control cells (p<0.001; Fig 9C and 9D and S4 Fig). Similar to our ROS experiment results, we did not observe any change in the cell cycle profile of XXI treated cells compared to DMSO treated ones (24 or 48 hours post treatment; S4 Fig).

## Discussion

DCT is an enzyme mainly involved in melanin synthesis in melanosomes. The expression of melanosomes is restricted to melanocytes and retinal pigment epithelial (RPE) cells [45]. Besides its main role in melanin synthesis, DCT is also involved in 1) detoxification of the melanin precursors; 2) it is known to limit DNA damage caused by chemotherapeutic agents, X-ray or UVB and gamma irradiation in melanoma cells, and 3) it decreases cell’s sensitivity to oxidative stress by increasing cellular glutathione (GSH) levels [28, 36, 46–49]. In addition to these functions, a recent genome-wide siRNA screen in HeLa cells surprisingly indicated a role for DCT in HPV16 infection [37]. However, the details of how DCT is involved in HPV infection have not been investigated yet.

Here, we studied DCT’s potential role in HPV infection and found that DCT depletion (i) notably decreased infection in HaCaTs; (ii) did not impact viral binding and delivery of PsV to early endosomes; (iii) did not prevent TGN localization of PsV and EdU-labeled pseudogenome; (iv) increased ER localization of EdU-labeled pseudogenome; (v) resulted in increased ROS in HaCaTs; (vi) interfered with HPV infection in partially different ways compared to γ-secretase inhibition.

DCT is normally expressed in melanocytes and in RPE cells; however, we found that DCT protein was expressed also in HaCaTs (basal keratinocytes) at a level comparable to RPE cells. Our findings on the effects of DCT knockdown on cells also suggest that the DCT protein is not only expressed but also functional in these cells.

In DCT depleted cells, viral particles were able to traffic through endosomes and the TGN; but not ER, where we saw increased localization at two different time points. This suggests that: (i) viral particles cannot exit from the ER; (ii) viral particles cannot localize to the nucleus; or (iii) viral particles traffics to another organelle and get degraded.

An earlier study has suggested that viral genome and L2 might localize from the ER to the nucleus during final stages of the cell division, when nuclear envelope was re-assembled following mitosis and ER acted as a source for membrane material required for nuclear envelope [22]. It has been also shown that HPV requires nuclear envelope breakdown during mitosis to be able to localize to the nucleus [24]. In our experiments, we observed increased viral DNA localization at the ER but an overall decrease in infection in DCT knockdown cells. This suggests viral DNA might be retained in the ER in DCT knockdown cells due to halted cell cycle, thus viral particles are incapable of further movement towards the nucleus.

It is known that oxidative stress might cause DNA damage and alter cell cycle progression in cells. To test whether increased localization of viral DNA in the ER is related to this phenomenon, we checked the oxidative stress levels of DCT knockdown and control cells and we, indeed, observed higher levels of ROS in DCT knockdown cells. Based on this observation, we then assessed whether increased ROS levels would cause DNA damage in DCT knockdown cells. Utilizing two well-known markers of DNA damage, pH2A.X and pChk2, we found that DCT knockdown cells have higher levels of DNA damage compared to control cells.

Extensive DNA damage can lead to delayed cell progression or even cell death when the damage persists. In our experiments, DCT knockdown cells were always fewer than the control siRNA transfected cells even though we were using same number of cells to seed our experiments. Having observed increased ROS levels and DNA damage in DCT knockdown cells, we wanted to investigate whether DCT knockdown cells were either going under apoptosis at an increased rate or were proliferating slower due to altered cell cycle. To distinguish between these two cases, we looked for apoptosis markers in DCT knockdown and control cells by western blot and saw that the apoptosis markers were absent in both treatments. We, then, investigated the cell cycle progression of DCT knockdown and control cells by two independent methods: a) using phosphorylated Rb antibody as our marker for cell cycle progression, we found that pRb levels in DCT knockdown cells are less than half of control cells’ pRb levels; b) we also measured the DNA content of DCT knockdown and control cells by PI staining and analyzed the cell cycle profiles under each treatment. The fraction of cells that were at G0/G1 phase was significantly higher in DCT knockdown cells compared to control cells. Although we did not detect a complete cell cycle arrest in DCT knockdown cells, their cell cycle profile was significantly altered compared to control cells, which might explain the relatively smaller number of cells in DCT knockdown experiments compared to normal cells. However, further studies are needed to assess whether a higher-efficiency DCT knockdown would alter cell cycle in a more drastic way or whether antioxidant treatments can rescue infection in DCT knockdown cells.

In a study focusing on the effect of XXI on HPV infection, Zhang and colleagues have reported that XXI treatment prevents vesicular trafficking of HPV to the TGN and subsequently to the ER [22]. Authors suggested that Golgi entry by HPV16 PsV requires γ-secretase cleavage of a transmembrane vesicle protein involved in endosome to Golgi transport. Our results in this study, where we observed PsV localization to TGN and ER in the XXI-treated cells, differ from these previous findings. Given that we and Zhang et al. both used HaCaT cells, used similar PsV constructs, antibodies and the same γ-secretase inhibitor in the experiments, the difference in our observations may be attributed to the different concentrations of the inhibitor used: in our study, we used XXI at 300pM concentration, whereas Zhang et al used the inhibitor at 250nM, approximately 1000 fold higher concentration than ours. The higher concentration of the drug may have relatively more drastic effects on the cells, and thus it is likely that the virus might not be able to localize in Golgi and ER under stronger γ-secretase inhibition. Another difference is that we treated the cells with XXI at the time of the infection and did not wash off the media with the inhibitor; whereas in the other study, they pretreated the cells for 1 hour with the inhibitor at 37°C before infection and it is not clear if they washed off media with the inhibitor.

In summary, our study provides evidence that DCT plays an important role in HPV16 infection in HaCaTs. In our hands, a partial DCT knockdown led to a significant decrease in PsV infection. Furthermore, we suggest that in the absence of DCT, increased ROS and DNA damage affect cell cycle progression, which hinders viral DNA’s egress from ER and localization to nucleus—an event required for successful infection. Our comparisons of DCT depletion to γ-secretase inhibition were not conclusive enough to suggest that DCT-mediated γ-secretase function is crucial for HPV infection. Therefore, better characterization of the relationship between DCT and its cleavage by γ-secretase in the context of HPV infection requires further investigation.

## Supporting Information

S1 Fig Efficiency of HPV16 PsV infection of HaCaT cells in the presence of increasing concentrations of XXI. Infection was quantified by flow cytometry. Error bars show the standard deviation of three experimental replicates in which 10,000 cells were analyzed for GFP expression to obtain the percent of infected cells.
(EPS)

S2 Fig Colocalization of TGN with the viral capsid is not affected by XXI or DCT siRNA treatment of HaCaTs at 16hpi. (A-H) DMSO, XXI, control siRNA or DCT siRNA treated HaCaTs were infected with HPV16 PsV. TGN was stained with TGN-46 (green), the viral capsid was stained with H16.V5 (red) and the nuclei were stained with DAPI (grey). Colocalization of the viral capsid and TGN appears yellow. (I and J) The JACoP plugin for ImageJ was used to measure the M1 coefficient (fraction of red signal overlapping with green signal) with three confocal scans for each condition.
(EPS)

S3 Fig DNA damage is increased in DCT knockdown cells. (A) Cell lysates from control and DCT knockdown cells were harvested on the day of the knockdown and subjected to western blotting. Western blot results of two membranes are shown with their loading control, actin. (B) pChk2 band intensities of each lane were quantified and normalized first against the corresponding actin or Chk2 measurements and then against the normalized pChk2 levels in control samples. The bar graph shows the normalized pChk2 levels (against actin or Chk2) of DCT knockdown cells compared to their controls.
(EPS)

S4 Fig Cell cycle profiles of DCT knockdown and XXI treated cells with their corresponding control treatments. (EPS)

S1 Table Cell count results of DCT and control siRNA treated HaCaTs. (PDF)
