# Supplementary material for: The Role of DCT in HPV16 Infection of HaCaTs
Source: PLoS One. 2017 Jan 17;12(1):e0170158. doi: 10.1371/journal.pone.0170158 (PMC5240977; doi:10.1371/journal.pone.0170158)

**Table S1** HaCaT cells were plated on 12-well-plates at equal densities for each experiment. The next day, cells were transfected with control or DCT siRNA. siRNA transfection was repeated on the following day. 72 hours after first transfection, the cells were stained with trypan blue and counted using a hemocytometer.

| | siRNA | Number of cells |
|---|---|---|
| Experiment 1 | Control | 2620000 |
| | DCT | 1500000 |
| Experiment 2 | Control | 1920000 |
| | DCT | 1100000 |
| Experiment 3 | Control | 2680000 |
| | DCT | 1000000 |
| Experiment 4 | Control | 6840000 |
| | DCT | 3600000 |
| Experiment 5 | Control | 4200000 |
| | DCT | 1260000 |

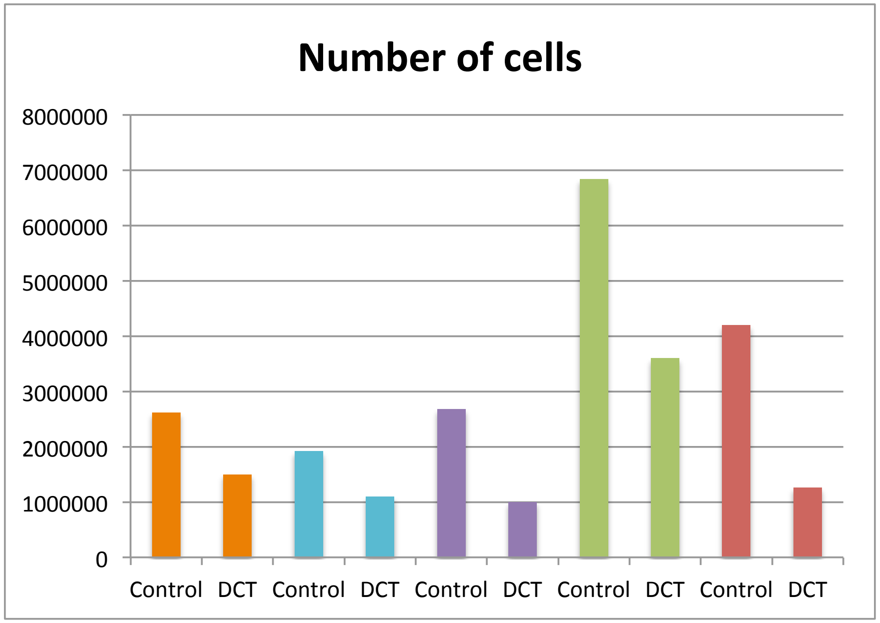

Supplement: S1 Table — (PDF) [file pone.0170158.s005.pdf]
